# Breastfeeding media coverage and beliefs during the COVID-19 pandemic in Mexico: implications for breastfeeding equity

**DOI:** 10.1186/s12939-021-01588-y

**Published:** 2021-12-20

**Authors:** M. Vilar-Compte, P. Gaitán-Rossi, E. C. Rhodes, V. Cruz-Villalba, R. Pérez-Escamilla

**Affiliations:** 1 Department of Public Health, Montclair State University, University Hall 4157, 1 Normal Ave, Montclair, NJ 07043 USA; 2 Yale School of Public Health, 135 College St, New Haven, CT 06510 USA; 3 Yale School of Medicine, 333 Cedar St, New Haven, CT 06510 USA; 4 Research Center for Equitable Development EQUIDE, Universidad Iberoamericana, Prolongación Paseo de la Reforma 880, Lomas de Santa Fe, 01219 Mexico City, Mexico

**Keywords:** Breastfeeding, COVID-19, Infant feeding, Inequities, Media analysis

## Abstract

**Background:**

Because breastfeeding offers short- and long- term health benefits to mothers and children, breastfeeding promotion and support is a public health priority. Evidence shows that SARS-CoV-2 is not likely to be transmitted via breastmilk. Moreover, antibodies against SARS-CoV-2 are thought to be contained in breastmilk of mothers with history of COVID-19 infection or vaccination. WHO recommends direct breastfeeding as the preferred infant feeding option during the COVID-19 pandemic, even among women with COVID-19; but conflicting practices have been adopted, which could widen existing inequities in breastfeeding. This study aims to describe how information about breastfeeding was communicated in Mexican media during the pandemic and assess Mexican adults’ beliefs regarding breastfeeding among mothers infected with COVID-19.

**Methods:**

We conducted a retrospective content analysis of media coverage on breastfeeding in Mexico between March 1 and September 24, 2020, excluding advertisements. For the content analysis, we performed both a sentiment analysis and an analysis based on strengths, weaknesses, opportunities, and threats (SWOT) for breastfeeding promotion. Additionally, we conducted a descriptive analysis of nationally representative data on adults’ beliefs about breastfeeding from the July 2020 round of the ENCOVID-19 survey in Mexico and stratified the results by gender, age, and socioeconomic status.

**Results:**

A total of 1014 publications on breastfeeding were identified on the internet and television and in newspapers and magazines. Most information was published during World Breastfeeding Week, celebrated in August. The sentiment analysis showed that 57.2% of all information was classified as positive. The SWOT analysis indicated that most information focused on current actions, messages, policies, or programs that enable breastfeeding (i.e., strengths) or those not currently in place but that may enable breastfeeding (i.e., opportunities) for breastfeeding promotion. However, ENCOVID-19 survey results showed that 67.3% of adults living in households with children under 3 years of age believe that mothers with COVID-19 should not breastfeed, and 19.8% do not know whether these mothers should breastfeed. These beliefs showed differences both by gender and by socioeconomic status.

**Conclusions:**

While the Mexican government endorsed the recommendation on breastfeeding during the COVID-19 pandemic, communication was sporadic, inconstant and unequal across types of media. There was a widespread notion that mothers with COVID-19 should not breastfeed and due to differences on beliefs by socioeconomic status, health inequities could be exacerbated by increasing the risk of poorer breastfeeding practices and preventing vulnerable groups from reaping the short and long-term benefits of breastfeeding.

**Table:** 

This article is a part of the Interventions and policy approaches to promote equity in breastfeeding collection, guest-edited by Rafael Pérez-Escamilla, PhD and Mireya Vilar-Compte, PhD	

## Background

The protection, promotion, and support of breastfeeding is a priority for public health, since breastfeeding offers mothers and children a constellation of short- and long-term health benefits [1, 2]. Evidence has consistently shown that the severe acute respiratory syndrome coronavirus 2 (SARS-CoV-2) is not likely to be transmitted via breastmilk (i.e., vertical transmission) [3–11]. In addition, COVID-19 appears to be less prevalent and, generally, less severe in infants [12, 13]. There is evidence that breastmilk from mothers with a history of COVID-19 infection contains antibodies against SARS-CoV-2, including Immunoglobulin A (IgA) and Immunoglobulin G (IgG) [12, 14–19]. It has been hypothesized that these antibodies may offer an infant protection against COVID-19, though more research – including studies with larger samples and longer follow-up periods – is needed to confirm this, since the magnitude, functionality, and durability of the breastmilk immune response is still unknown [15]. Furthermore, prospective cohort studies have found that anti-SARS-CoV-2 IgA and IgG generated by anti-COVID-19 mRNA-based vaccines administered to lactating and pregnant mothers are transferred to their babies via breastmilk [14, 20, 21] and umbilical cord blood [22, 23], while COVID-19 mRNA is not transferred [14, 24, 25].

Breastmilk contains anti-microbial and anti-inflammatory factors that promote the development of the immune system and protects them from pneumonia, diarrhea, and other poor health conditions [26, 27]. Accounting for this evidence, and the short and long-term harm resulting from the separation of the mother-child dyad during the perinatal period, as well as the susceptibility of newborns to person-to-person spread of COVID-19 through contact with mothers and caregivers (i.e., horizontal transmission) [12], the World Health Organization (WHO) recommends that women with COVID-19 should breastfeed their babies [10, 12, 28] and that feeding directly from the breast, referred to as direct breastfeeding, should be supported as the preferred recommended infant feeding option during the pandemic. Accordingly, mothers with confirmed or suspected COVID-19 should be informed about the importance of continued direct breastfeeding. During the birth hospitalization period, mother-infant dyads should be cared for together, including skin-to-skin contact and room sharing, which is critical for helping mothers establish and continue breastfeeding [13]. Separating mothers and their infants during this time increases neonatal morbidity and mortality [29].

Despite the WHO’s strong emphasis on promoting breastfeeding and keeping the mother-infant dyad together in the hospital immediately after birth during the COVID-19 pandemic, some governments, professional organizations, and hospitals have not adopted these practices [30]. During the early stages of the pandemic, recommendations against practices supportive of breastfeeding were common, even in countries with high infant mortality rates [31]. For example, some governments and health care systems recommended that infected mothers be separated from their infants after birth to reduce the risk of infant COVID-19 infection. Inconsistencies in guidance from reputable international and professional agencies such as the Centers for Diseases Control and Prevention (CDC) in the United States and medical associations such as the American College of Obstetricians and Gynecologists (ACOG) may have contributed to the misalignment with WHO recommendations in countries and healthcare facilities. Moreover, such recommendations generated uncertainty about breastfeeding among new parents [13, 32]. Sola et al. analyzed evidence from 7 countries of the Ibero American Society of Neonatology (Argentina, Colombia, Ecuador, Equatorial Guinea, Honduras, Peru and Dominican Republic) from March to May 2020 to evaluate how the pandemic impacted pregnant and breastfeeding women and newborns in Latin America [33]. Findings showed that lack of breastfeeding support and mother-infant dyad separation among COVID-19 positive women were common during the early months of the pandemic, as well as increased practices of formula feeding.

Furthermore, despite the emerging evidence suggesting that it is safe to breastfeed after receiving the COVID-19 vaccine [34] and that doing so may benefit both mothers and infants [23], initial vaccination policies denied breastfeeding women’s access to the vaccine. These policies were created due to possible safety concerns, which could not be ruled out because breastfeeding women were not included in vaccine trials. Indeed, some federal and local governments and COVID-19 vaccination centers in different countries [34–36], initially required consent forms or disseminated fact sheets stating that mothers who receive the COVID-19 vaccine should not breastfeed or mothers should contact their healthcare providers to further discuss the safety of the vaccine during breastfeeding. This guidance may have led healthcare providers to not promote and support breastfeeding and may have discourge mothers not to breastfeed their infants [37], even after such guidance was later reversed [38].

Diverse pre-COVID socio-cultural beliefs about breastfeeding, the uncertainty and social anxiety brought by the pandemic [12], and the marketing strategies from the breastmilk susbtitutes industry during the pandemic might have also prompted healthcare providers and mothers to not start or discontinue breastfeeding during the pandemic. Rising mis and dis-information regarding health could have added to the problem. A multi-institutional statement highlighted that the pandemic has produced an infodemic – an overabundance of information, both online and offline, that includes deliberate attempts to disseminate wrong information to undermine the public health response and advance alternative agendas [39]. It is therefore critical to track the extent to which official health authorities’ messages during the pandemic spread among the population, as well as the scientific validity of their messages [40]. A previous study found that 6% of tweets about breastfeeding and COVID-19 that were tweeted up until March 27, 2020 contained scientifically unfounded recommendations or information tailored for commercial use that undermine breastfeeding, such as promotion of breast pumps and feeding bottles [40]. Media can be a powerful tool to promote or discourage breastfeeding not only through dissemination of information, but also as a mechanism for generating public interest and legitimizing a problem [41]. Given its influence, media helps determine which policy issues are most salient to the public [42], and, thus, influence the formation of public agendas [43] and the “public mood” towards a given issue such as breastfeeding during the pandemic. There is little information on media coverage on breastfeeding practices and COVID-19 and its influence on breastfeeding behaviors and beliefs.

Separating mothers from their infants during the birth hospitalization and suggesting mothers not initiate or discontinue breastfeeding to reduce the risk of COVID-19 in infants may widen existing inequities in breastfeeding. Socio-economically marginalized groups are least able to stay home during lockdowns and adhere to social distancing guidelines to reduce the spread of COVID-19, due to the nature of their jobs [44]. These groups are, therefore, least able to minimize their viral exposure. They might also be more likely to suffer the adverse consequences of mother-infant separation during the birth hospitalization, and in turn, to face barriers to breastfeeding [45]. Furthermore, vulnerable groups have also suffered disproportionately from the adverse economic consequences of the pandemic, such as job loss and furlough [44]. Hence, breastfeeding in the context of COVID-19 is of increased importance to mitigate household food insecurity, since breastmilk is the most affordable form of infant and young child nutrition and the high cost of BMS can further strain household budgets among families already facing financial stress and reduce funds available for food expenditure [13]. It is also well established that breastmilk provides the cleanest and safest form of infant and young child nutrition during crises, and it is the normative standard for infant nutrition [3]. Hence, breastfeeding should be considered as a fundamental protective and health promotion measure for infants during the pandemic [27].

Mexico has been severely affected by the COVID-19 pandemic; it has the fourth highest number of COVID-19 related deaths in the world [46]. The public health emergency in Mexico has had sustained negative effects on household income, employment, food insecurity, and mental health since the beginning of the pandemic in March 2020 [47–49]. Prior to the pandemic, there had been a steady increase in breastfeeding rates in Mexico. For example, the exclusive breastfeeding rate among infants under six months improved from 14.4% in 2012 to 28.3% in 2018 [50, 51]. Due to the COVID-19 pandemic, however, these improvements may slow down and widen breastfeeding inequities if actions to promote and protect breastfeeding during this public health emergency are not taken. While the Mexican government [52], the national institutes of pediatrics and perinatology [53], and the Mexican Association of Pediatricians [54] adhered to scientific evidence and WHO guidance on breastfeeding during the COVID-19 pandemic early in the pandemic and established guidelines and pronouncements accordingly, it is unlcear if these guidelines and prouncements were adequately disseminated and implemented. Due to changes in the Federal administration prior to the pandemic, the *National Strategy of Breastfeeding* [55] had expired and not been renewed. Thus, government commitment and investment in breastfeeding protection, promotion, and support was lacking when the pandemic emerged.

The aims of this study were (a) to describe how Mexican government recommendations on breastfeeding during COVID-19 were communicated in traditional media platforms, including newspaper, magazine, radio, and television, (b) to assess Mexican adults’ beliefs regarding breastfeeding among mothers infected with COVID-19, and (c) to assess if there were differences in these beliefs by socioeconomic status, with the goal of understanding whether adults who are socio-economically disadvantaged might be more likely to hold views that do not align with current scientific recommendations on breastfeeding compared with adults who are not.

## Methods

The study used two complementary methodological approaches: (a) a retrospective content analysis of media coverage on breastfeeding in print and online media sources in Mexico between March 1 and September 24, 2020 and (b) a descriptive analysis based on nationally representative data from a telephone survey, the Survey on COVID-19 Effects on Wellness in Mexican Households (ENCOVID-19, for its acronym in Spanish).

### Analysis of media coverage on breastfeeding

The analysis included any newspaper, magazine, radio, and television coverage of breastfeeding. Social media was excluded from the analysis because analyzing it requires a different methodological approach. The media search was conducted by *Eficiencia Informativa*, a company that specializes in media monitoring and content analysis. It was done using a combination of the following key words in Spanish: breastfeeding, maternity leave, Becoming Breastfeeding Friendly (a global initiative to promote, protect, and support breastfeeding in hospitals), and Permanent Interinstitutional Support Group for Breastfeeding (a group led by the Ministry of Health). The inclusion criteria for the media search were: (a) date of publication between March 1 and September 24, 2020; (b) includes information on breastfeeding; (c) published and disseminated in Mexico, and (d) not an advertisement.

The information identified in the media search was organized both by media platform and by date, in order to identify the frequency of documents or programs (i.e., TV and radio) mentioning breastfeeding per month. A thematic analysis was performed, in order to organize the information into key themes. These themes were then analyzed through both a sentiment analysis and an analysis that identifies strengths, weaknesses, opportunities, and threats, which is commonly referred to as a SWOT analysis.

Through the sentiment analysis, each piece of information was classified as positive, neutral, or negative with regards to its potential impact on breastfeeding promotion during the pandemic; examples of such classification can be found in Table 1.

**Table 1:** Examples of positive, neutral, and negative sentiment classification for breastfeeding promotion

Positive information	Neutral information	Negative information
Recommendations to continue breastfeeding during the pandemic	Reports on the importance of traditional midwifery in indigenous communities during the pandemic	Reports of increase marketing of breastmilk substitutes during the pandemic
Reports of no evidence that COVID-19 can be transmitted via breastmilk	Reports on decrease in Mexican women’s fertility rate	Reports of detection of COVID-19 in breastmilk
Promotion of breastfeeding continuation during social distancing and lockdown measures by the Mexican Ministry of Health	Reports on the first births from Mexican mothers infected with COVID-19	Reports about health professionals’ influence on the discontinuation of breastfeeding among mothers

The SWOT analysis was based on a breastfeeding media analysis methodology previously used in Mexico [56]. The SWOT analysis examined organizational and environmental strengths and weaknesses, opportunities for improvement, and potential threats to breastfeeding promotion during the pandemic. This sort of analysis intends to inform future actions to improve breastfeeding promotion and communication. Two of the authors (MVC, VCV) independently verified the classification of doucments and programs performed by *Eficiencia Informativa* and generated a SWOT matrix. The operational definitions for each category of the SWOT analysis is presented in Table 2.

**Table 2:** SWOT operational definitions and examples of classification of information based on this analysis

Category	Operational definition^a^	Example
Strengths	Current actions, messages, policies, or programs that enable breastfeeding	The Mexican Ministry of Health promoting breastfeeding continuation during the COVID-19 pandemic
Weaknesses	Actions, messages, policies, or programs currently in place that negatively affect breastfeeding	Newborns separated from their mothers during the birth hospitalization due to fear of virus transmission from mothers to newborns.
Opportunities	Actions, messages, policies, or programs not currently in place that may enable breastfeeding	United Nations Children’s Fund (UNICEF) Mexico and the Mexican government initiating a new Cooperation Program for the 2020–2025 period.
Threats	Actions, messages, policies or programs not currently in place that may negatively affect breastfeeding	The COVID-19 pandemic projected to have collateral effects on health, not directly associated to the virus transmission, such as reduction in breastfeeding rates, delay in diagnoses, and mental health issues

^a^Adapted from Ferré-Eguiluz et al. [56]

### Descriptive analysis of ENCOVID-19 data

The ENCOVID-19 is a monthly telephone survey that was started in April 2020 to document the consequences of the COVID-19 pandemic in Mexico. With a repeated cross-sectional design, the ENCOVID-19 is administered monthly to a nationally representative sample of individuals 18 years and older who have a mobile phone. Therefore, post-stratification sampling weights were used to correct minor deviations from Mexico’s demographic structure.

The ENCOVID-19 survey included two questions on breastfeeding in the July 2020 round. The sample included 1584 individuals and was representative of households with children under 18 years of age. Amongst them, 279 people lived in a household with a child under 3 years old. Participants were asked to respond to the following two questions: “From what you know, if a mother has COVID-19, should she breastfeed?” Response options were Yes, No, and Don’t know. If they answered no, the follow-up question was: “What is the reason not to breastfeed?”. Response options were: i) The virus is transmitted through breastmilk; ii) My physician recommended not to; and iii) The mother should be isolated. Although breastfeeding practices are most relevant for this subpopulation, the survey did not capture the perceptions of the general population, which is also exposed to media messages about breastfeeding. Moreover, the respondent of the survey is the person who answered the call and not necessarily the caregiver in the household.

In addition to collecting data on demographic characteristics (sex, age in years), the ENCOVID-19 collects data for calculating the Mexican Association of Market and Opinion Intelligence Agencies (AMAI, for its acronym in Spanish) index [57], an assets-based household socioeconomic index with six indicators, including: (i) education level of the head of household; (ii) number of bathrooms; (iii) number of cars or vans; (iv) having an Internet connection; (v) number of household members 14 years or older who are working, and (vi) number of bedrooms.

Descriptive analyses were conducted to generate frequencies and proportions of responses to each question on breastfeeding by gender, age, and socioeconomic status.

## Results

### Analysis of media coverage on breastfeeding

**Fig. 1:**
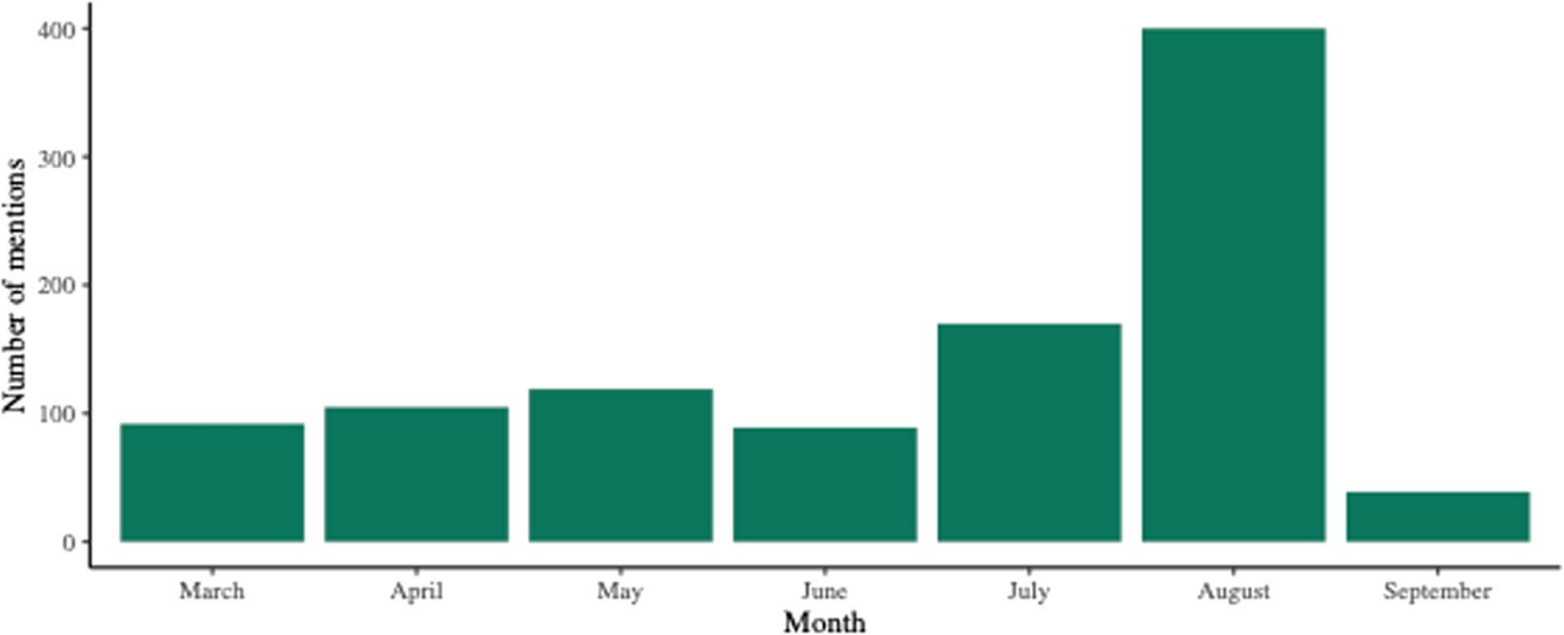
Total mentions of breastfeeding in the context of COVID-19 in the media by month from March to September 2020

A total of 1014 mentions of breastfeeding in the context of COVID-19 were identified, with most mentions on the Internet (83.6%) followed by newspapers (10.4%), television (3.6%), magazines (1.7%), and radio (0.8%) (Table 3). During August 2020 when World Breastfeeding Week was celebrated in Mexico, these mentions spiked, reaching 400 during the entire month (Fig. 1).

**Table 3:** Mentions of breastfeeding in the context of COVID-19 classified by type of media

Type of media	Mentions, n (%)
Internet (online versions of newspapers and magazines)	848 (83.6%)
Newspapers	105 (10.4%)
TV	36 (3.6%)
Magazines	17 (1.7%)
Radio	8 (0.8%)

**Fig. 2:**
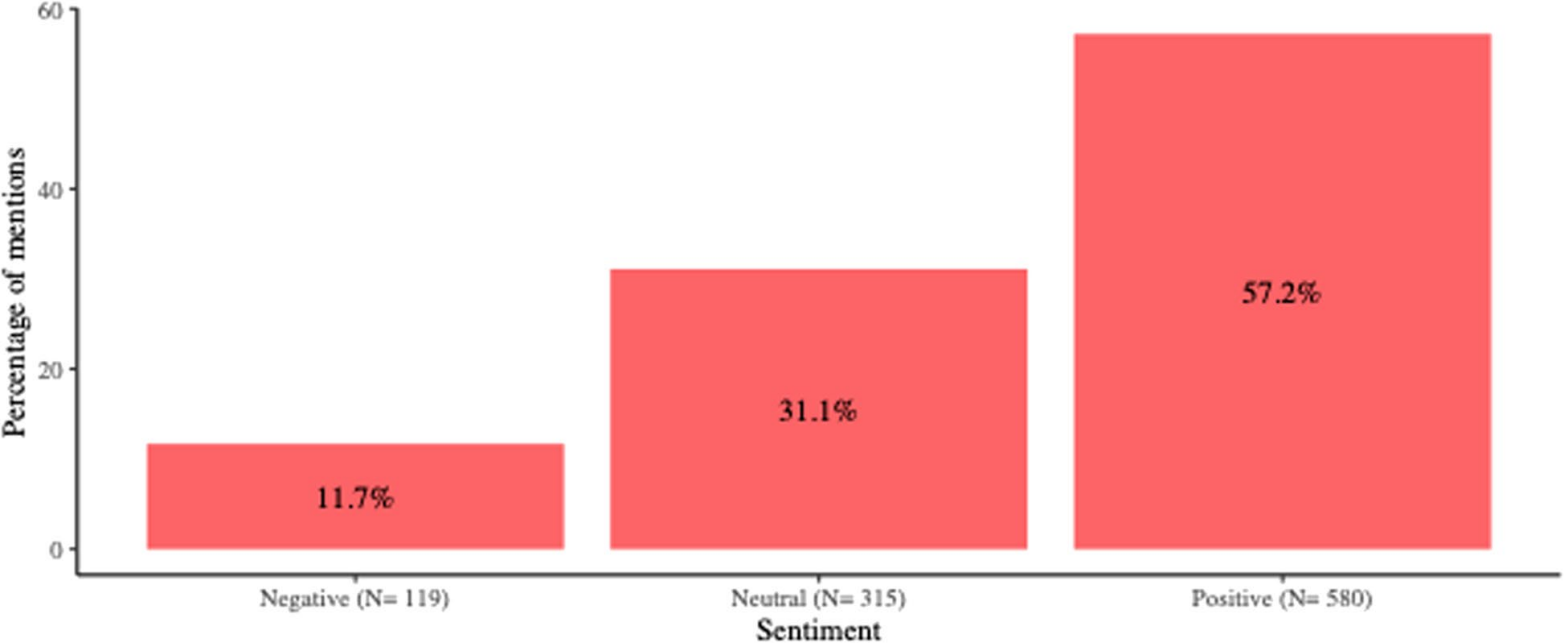
Breastfeeding sentiment analysis in the context of COVID-19 from March to September 2020

More than half of all mentions were classified as positive for breastfeeding promotion (Fig. 2), and in all media platforms, positive information was higher than negative and neutral information combined, except for information published in magazines, where most of the information on breastfeeding was classified as neutral. It is noteworthy that messages during World Breastfeeding Week in August were more likely to be of a positive nature than messages at other times of the year. In fact, during August 2020 only 5% of the messages were negative, while 19.75% were neutral and 75.25% were positive.

The most prevalent negative theme was the increase of marketing of breastmilk substitutes during the pandemic, informed through news reports about it found on the analyzed media platforms, accounting for 67 (6.6%) of total mentions of breastfeeding. Six (0.6%) mentions stated that COVID-19 was detected in breastmilk and 4 (0.4%) mentions referred to the influence of advice from medical doctors in women’s decisions not to breastfeed. Positive themes on breastfeeding are shown in Table 4.

**Table 4:** Positive themes found about breastfeeding and COVID-19

Theme	Mentions, n (% of total)
Recommendations to keep breastfeeding during the pandemic	90 (8.9%)
Reports of no evidence that COVID-19 could be transmitted via breastmilk	51 (5%)
Announcement that the Mexican Ministry of Health would promote breastfeeding during social distancing and lockdown measures	29 (2.9%)
Feeding recommendations during the pandemic made from United Nations (UN) agencies to the Mexican government	12 (1.2%)

In accordance with the findings from the sentiment analysis, the SWOT analysis results showed that almost all of the information (84.6%) promoted breastfeeding; 64.3% of all mentions were classified as strengths and 20.3% as opportunities, whereas 11.7% of them were classified as threats and only 3.6% as weaknesses. Results from the SWOT analysis are shown in Table 5.

**Table 5:** Results from the SWOT^a^ analysis

**Strengths**	• Follow-up of the World Breastfeeding Week’s activities • Recommendations to keep breastfeeding during the pandemic • Mexican Ministry of Health’s efforts to promote breastfeeding during the pandemic • Comments about the importance of breastfeeding from Mexican Ministry of Health’s and UNICEF’s experts
**Weaknesses**	• Lack of female representation in decision-making at the policy level and in breastfeeding events • Lack of meaningful public policies to protect breastfeeding • Separation of mother-infant dyads during the birth hospitalization period during the COVID-19 pandemic
**Opportunities**	• UN agencies’ general nutrition recommendations during the pandemic, and breastfeeding highlighted as a measure to combat food vulnerability and insecurity • New Cooperation Program between UNICEF Mexico and the Mexican government for the 2020–2025 period, where breastfeeding protection and promotion can be incorporated • Non-governmental organizations’ demands to regulate BMS donations during the pandemic, which were promoted by pharmacies and BMS companies to the general public
**Threats**	• Increase of marketing of BMS during the pandemic^b^ • Employment and household work inequities during lockdown measures (i.e., women taking a disproportionate role in caring for children) • Increasing C-section rates, which are associated with reduced breastfeeding • Influence of medical doctors on women’s decisions not to breastfeed

^a^SWOT refers to strengths, weakness, opportunities, and threats

^b^Evidence of marketing was found through an examination of documents/programs referring to promotional marketing materials, but not through an assessment of advertisments

### Descriptive analysis of ENCOVID-19 data

**Fig. 3:**
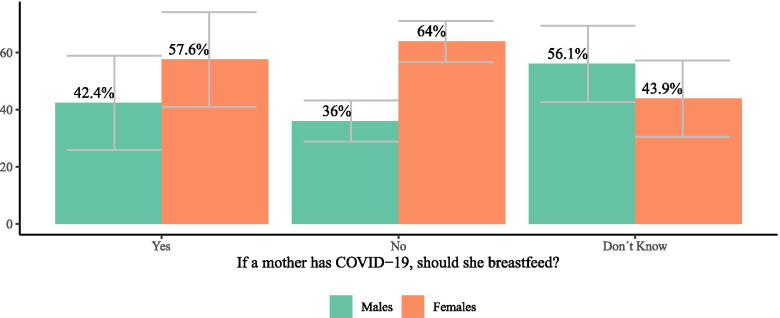
Beliefs about breastfeeding during the COVID-19 pandemic among adults living in households with children 3 years and younger (ENCOVID-19- July 2020. *N* = 279), by sex. *Bars represent 95% confidence intervals

Our analysis of ENCOVID-19 survey data found that 67.3% of people living in households with children under 3 years of age believed that mothers with COVID-19 should not breastfeed, and 19.8% reported that they did not know if mothers with COVID-19 should breastfeed. Notably, the proportion of women who thought mothers should not breastfeed was higher than the proportion of men with this belief. Furthermore, more men reported that they did not know if mothers with COVID-19 should breastfeed than women (see Fig. 3). Beliefs about breastfeeding during the COVID-19 pandemic did not differ according to age.

**Fig. 4:**
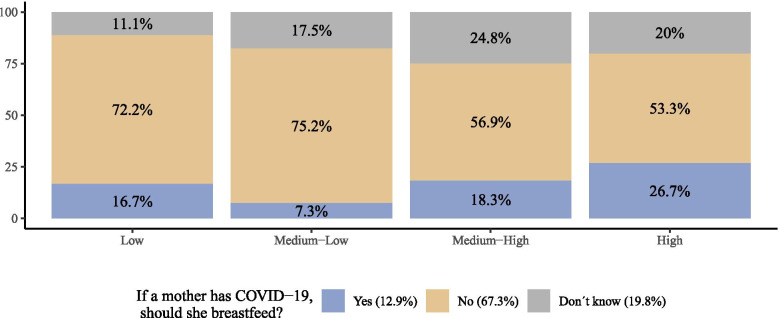
Beliefs of breastfeeding during the COVID-19 pandemic among adults living in in households with children 3 years and younger (ENCOVID-19- July 2020. *N* = 234), according to socioeconomic status

A larger proportion of adults living in households with low and medium-low socio-economic status believed that mothers with COVID-19 should not breastfeed (72.2 and 75.2%, respectively) compared with adults living in households with medium-high and high socio-economic status (56.9 and 53.3%, respectively) (Fig. 4). Among adults from households with high socioeconomic status, only a minority of them believed that mothers with COVID-19 should breastfeed (26.7%). The proporition of adults who did not know the answer ranged from 11% in households with low socioeconomic status to 24.8% in households with medium-high socioeconomic status (Table 6).

**Table 6:** Beliefs about breastfeeding during the COVID-19 pandemic among adults living in households with children 3 years and younger (ENCOVID-19- July 2020. *N* = 279), according to socio-economic status

	No	Yes	Don’t Know	Total
Low	72.2% (13)	16.7% (3)	11.1% (2)	18
Medium-Low	75.2% (103)	7.3% (10)	17.5% (24)	137
Medium-High	56.9% (62)	18.3% (20)	24.8% (27)	109
High	53.3% (8)	26.7% (4)	20% (3)	15
	186	37	56	279

*Note*: Socioeconomic status group differences by response to COVID-19 are statistically significant (Kruskal-Wallis chi-squared = 8.7655, df = 3, *p*-value = 0.033). Post-hoc analyses with the Holm adjustments indicate that the difference between medium-low and medium-high is statistically significant (*p* = 0.037). Fisher’s exact test also indicated a statistically significant association (chi-squared = 13.893, df = 6, *p*-value = 0.031)

Most adults who believed that women infected with COVID-19 should not breastfeed, reported that the reason why mothers should not do so was because “the virus is transmitted by milk” (62.4%). About a quarter ((23.7%) of adults reported that the “mother should be isolated.” A small proportion of adults reported that their “physician recommended not to” (1.6%), while 12.4% said their belief was due to “Other” reasons.

## Discussion

The COVID-19 pandemic is posing challenges to breastfeeding. Evidence supports the continuation of direct breastfeeding, skin-to-skin contact, and not separating the mother-infant dyad during the birth hospitalization even if mothers are infected by SARS-CoV-2. Despite such evidence, it has been hard to combat the public’s unsubstantiated fears of infection that arose due to initial inconsistencies in guidance on breastfeeding during the pandemic. In addition, the early controversy regarding the inclusion of pregnant and breastfeeding women in the COVID-19 vaccination schemes could have also affected breastfeeding decisions.

This study offers important lessons from Mexico, which might be relevant for other countries. First, as shown by our analysis of media coverage, traditional media platforms (i.e., magazines, newspapers, TV, radio) disseminated messages released by the government and health organizations experts backing the recommendations of the WHO to directly breastfeed infants even if mothers test positive for SARS-CoV-2. The messages from experts were mainly derived from press conferences, interviews, and governmental reports and guidelines that were subsequently disseminated by media.

Second, despite the recognition and adherence to WHO recommendations by the government and key professional bodies, the media analysis revealed that the promotion of such messages was unevenly distributed by type of media platform. This finding is important because women from different socioeconomic backgrounds and educational levels may be exposed to different media platforms. While almost all of the information regarding breastfeeding was distributed in online versions of traditional media (newspaper, magazines, radio and television), access to internet is deeply unequal by type of location (i.e., urban or rural), gender, education level, occupation, and socioeconomic status in Mexico [58]. There is a difference of 70.7 percentage points in access to internet connection between households with high and low socioeconomic status in Mexico. The gap in access to public television and radio is far smaller, with a difference of only 13.9 and 8.8 percentage points, respectively. Although public television is the most used and evenly distributed type of media in the country, most information on breastfeeding was found in online versions, which has the widest distribution gap by socioeconomic status. Therefore, vulnerable households with poor access to internet may be less likely to receive such information. This study also revealed an imbalanced distribution of messages across time, as most messages were distributed during World Breastfeeding Week in August; during this period a high number of positive messages were disseminated. The World Breastfeeding Week is a communication opportunity, but if dissemination is not continuous throughout the year, breastfeeding is at risk of losing salience in the public agenda and public opinion. Informing families about infant feeding choices amidst a pandemic, probably requires a more constant strategy.

Third, the media analysis showed the presence of marketing strategies of BMS during the COVID-19 pandemic. This finding is consistent with findings from studies in other regions during the same period, which show that BMS companies are capitalizing on fear of breastfeeding during the pandemic to market their products [59]. Such strategies to promote and distribute BMS during emergencies such as earthquakes [60, 61] and tsunamis [62] have been documented internationally [63]. Together, this evidence highlights that governments and international organizations need to step in to regulate BMS donations and guard mothers and infants against unnecessary promotion and distribution of BMS, which often reaches the most vulnerable populations. For this reason, the World Health Assembly urges all Member States to ensure evidence-based and appropriate infant and young child feeding during emergencies [64].

Lastly, results from our analysis of ENCOVID-19 data showed that most adults believed that an infant should not be breastfed if the mother is infected with COVID-19. Such beliefs were more common among lower socioeconomic groups, which could indeed lead to inequities in breastfeeding during a public health emergency. This is worrisome in light of the increased prevalence of food insecurity in Mexico during the pandemic, which has disproportionately affected lower socioeconomic status groups [47]. Decreases in breastfeeding rates can exacerbate household food insecurity due to the high costs of BMS. Furthermore, formula feeding is associated with increased morbidities in infants and young children. Prior studies have also documented increased risk of SARS-CoV-2 infection among low income households due to inequities in housing, employment, and transportation [65, 66]. Thus, pregnant and lactating mothers living in low income households may be at increased risk of infection, which in turn may imply and increase risk of infection that can discourage them from initiating and continuing breastfeeding.

Our findings indicate that breastfeeding promotion, protection, and support strategies must be prioritized by health professionals, the government, and advocacy groups targeting pregnant women and breastfeeding mothers of vulnerable socioeconomic groups in order to provide accurate information about breastfeeding during the pandemic to mothers and families. There is a need to implement evidence-based and consistent messaging through diverse types of media platforms, combined with strong breastfeeding protection, promotion, and support in clinics and hospitals. Governments are facing unprecedented challenges in providing health services amidst the pandemic, nonetheless, infant and young child feeding information and services during the COVID-19 pandemic need to be prioritized to avoid growing inequities in breastfeeding.

This study uses a novel mixed-methods approach that could be easily and rapidly deployed in other countries. The media analysis identified how breastfeeding recommendations were promoted and disseminated, as well as the limtiations of such process. The telephone survey captured beliefs about breastfeeding during the pandemic among members of families with children under 3 years of age. Our analyses documented breastfeeding promotion and belief gaps linked to barriers of media coverage and, potentially, to other determinants, including misinformation disseminated in social media, lack of adherence of health care providers to guidelines, and the normalization of formula feeding in the country. These determinants should be further studied.

Based on the findings of this study, there are four lessons that can inform communication around breastfeeding during future heath crises. First, it is important to use different media platforms to communicate messages about breastfeeding to mothers and caregivers. The messages need to be consistent and frequent; and, ideally, they should not only provide information but also potential solutions to face the challenges imposed by the crisis. In addition, messages and communication platforms should reach mothers and caregivers throughout all socioeconomic levels and cultural backgrounds. Second, scientific and evidence-based messages need to be communicated rapidly and efficiently to counteract misinformation. While the current study only focused on traditional media platforms, it is important to consider the role of social media in the rapid spread of mis/information that can affect the beliefs about breastfeeding in critical periods. A rapid response can also limit the impact of common promotions and donations of the BMS industry. Lastly, because health providers play a key role in informing mothers and caregivers about breastfeeding, communication strategies should also target them. For example, while the Mexican Government published clear guidelines that adhered to WHO recommendations, there were no targeted communication efforts to disseminate the key practices through traditional media platforms, hence, limiting the implementation at the clinical level. Finally, as highlighted in prior studies during crises and emergencies [60], governments need to have clearer protocols on how to enforce and monitor the protection and support of breastfeeding when such events arise.

This study had several limitations. First, the media analysis excluded social media (i.e. Twitter). Evaluating messages spread through social media could offer a different perspective on information about breastfeeding being disseminated during the COVID-19 pandemic. Such analyses require a different methodological approach than the one employed in this study, for example, network analysis. Second, the ENCOVID-19 sample is cross-sectional, precluding drawing causal inferences. Another limitation is that the media analysis was not able to capture the relative influence of messages in shaping beliefs. For example, during the pandemic a positive message about breastfeeding from the government might have had a different impact on beliefs than a negative message from stakeholders such as pediatricians, family members, or friends [67]. Furthermore, a limitation of the telephone survey is the limited coverage; in 2019, in Mexico, 89.4% had a mobile phone, but it decreased to 74% in rural areas [68]. In addition, we cannot ascertain if beliefs about breastfeeding might have changed throughout the pandemic. Future rounds of the ENCOVID-19 should include questions on beliefs about breastfeeding so that future studies can investigate whether and how these beliefs change over time.

## Conclusions

The COVID-19 pandemic has impacted families with infants and young children in many ways, among them, the decisions on how to feed them. In Mexico, the pandemic has dispproportionately affected infants and children from lower socio-economic households. The Mexican government publicly adhered and communicated the WHO recommendations on breastfeeding during the COVID-19 pandemic. Messages appeared in different types of media platforms. However, communication was sporadic, inconstant and unequal across different types of media. In addition, the media analysis revealed that while the government was promoting breastfeeding, there were other media messages linked to donations and promotion of BMS that violate the International Code of Marketing of Breastmilk Substitutes. The survey on breastfeeding beliefs during the pandemic among adults living in households with children under 3 years reveals the widespread notion that, if a mother is infected with COVID-19, she should not breastfeed. This belief was more prevalent among socio-economically disadvantaged families, which could be at higher risk of not initiating or continuing breastfeeding during the pandemic. This health inequity should be addressed through targeted actions. From a policy perspective, it is fundamental to consider that social inequities increase the risk of poorer breastfeeding practices and of not receiving the short and long-term health benefits of breastfeeding.

## Data Availability

The media analysis datasets used during the current study are available from the corresponding author on reasonable request. The ENCOVID-19 datasets used during the current study are available in the Zenodo repository, on the following link: 10.5281/zenodo.4602374.

## Abbreviations

AMAI: Mexican Association of Market and Opinion Intelligence Agencies (for its acronym in Spanish)
BMS: Breastmilk substitutes
ENCOVID-19: Survey on COVID-19 Effects on Wellness in Mexican Households (for its acronym in Spanish)
GILPALM: Permanent Interinstitutional Support Group for Breastfeeding (for its acronym in Spanish)
NGO: Non-governmental organization
SARS-CoV-2: Severe acute respiratory syndrome coronavirus 2
SWOT: Strengths, weaknesses, opportunities, threats
UN: United Nations
UNICEF: United Nations Children’s Fund
UK: United Kingdom
USA: United States of America
WHO: World Health Organization
